# Combined versus Single Perforator Propeller Flaps for Reconstruction of Large Soft Tissue Defects: A Retrospective Clinical Study

**DOI:** 10.3390/jpm12010041

**Published:** 2022-01-04

**Authors:** Amir K. Bigdeli, Oliver Didzun, Benjamin Thomas, Leila Harhaus, Emre Gazyakan, Raymund E. Horch, Ulrich Kneser

**Affiliations:** 1 Department of Hand, Plastic and Reconstructive Surgery, Burn Center, BG Trauma Center Ludwigshafen, Ludwig-Guttmann-Strasse 13, 67071 Ludwigshafen, Germany; oliver.didzun@googlemail.com (O.D.); benjamin.thomas@bgu-ludwigshafen.de (B.T.); leila.harhaus@bgu-ludwigshafen.de (L.H.); emre.gazyakan@bgu-ludwigshafen.de (E.G.); ulrich.kneser@bgu-ludwigshafen.de (U.K.); 2 Department of Hand and Plastic Surgery, University of Heidelberg, 69117 Heidelberg, Germany; 3 Department of Plastic and Hand Surgery, Friedrich-Alexander-University Erlangen-Nuremberg, Krankenhausstrasse 12, 91054 Erlangen, Germany; Raymund.Horch@uk-erlangen.de

**Keywords:** perforator propeller flap, combined perforator propeller flap, microsurgery, soft tissue reconstruction, propeller flap, perforator flap

## Abstract

Sufficient wound closure of large soft tissue defects remains a challenge for reconstructive surgeons. We aimed to investigate whether combined perforator propeller flaps (PPFs) are suitable to expand reconstructive options. Patients undergoing PPF reconstruction surgery between 2008 and 2021 were screened and evaluated retrospectively. Of 86 identified patients, 69 patients received one perforator propeller flap, while 17 patients underwent combined PPF reconstruction with multiple flaps. We chose major complications as our primary outcome and defined those as complications that required additional surgery. Postoperatively, 27 patients (31.4%) suffered major complications. The propeller flap size, the type of intervention as well as the operation time were not associated with a higher risk of major complications. A defect size larger than 100 cm^2^, however, was identified as a significant risk factor for major complications among single PPFs but not among combined PPFs (OR: 2.82, 95% CI: 1.01−8.36; *p* = 0.05 vs. OR: 0.30, 95% CI: 0.02−3.37; *p* = 0.32). In conclusion, combined PPFs proved to be a reliable technique and should be preferred over single PPFs in the reconstruction of large soft tissue defects at the trunk and proximal lower extremity.

## 1. Introduction

Modern reconstructive surgery offers a vast variety of surgical techniques for the reconstruction of soft tissue defects [1,2]. Although free flaps have been established as the standard procedure in the reconstruction of soft tissue defects, regional flaps might be used depending on the size and characteristics of a defect [3]. However, large defects often require complex solutions with multiple flaps or tissue expansion to ensure sufficient wound closure [4,5]. Frequently used combinations consist of muscle flaps, sliding flaps as well as rotation flaps, which are either combined with one another or with a free flap [6,7]. Nevertheless, limitations of regional flaps are due to arc of flap rotation, flap size to defect size ratio, wound infections, and donor-site morbidity while free flaps are limited whenever vessels for anastomosis are insufficient [8]. Furthermore, prior surgeries often lead to significant scarring and, hence, may impede the use of conventional regional flaps.

Since the introduction of perforator propeller flaps (PPFs), they have gained increasing popularity [9,10]. By the definition of the “Tokyo consensus” on propeller flaps, propeller flaps are “island flaps that reach the recipient-site through an axial rotation“ [11]. As a combination of a reliable pedicled flap along with low donor-site morbidity, PPFs offer high flexibility and, therefore, have led to versatile use. First employed in the reconstruction of the upper and lower extremity, PPFs have also become an established technique in the reconstruction of soft tissue defects of the trunk [12]. Even though PPFs have shown to be a reliable reconstructive option, there is a gap in the literature regarding the potential advantages associated with using combined PPFs instead of single PPFs (Table 1). This study aims to compare the outcome of single and combined PPFs, to determine the prevalence of complications among both techniques, and to assess the potential use of combined PPFs in the reconstruction of large soft tissue defects.

## 2. Materials and Methods

### 2.1. Patients

Medical records of all patients who received a PPF reconstruction surgery between 2008 and 2021 at the University Clinic of Erlangen Nuremberg and the BG Trauma Center Ludwigshafen were identified and evaluated retrospectively. All surgeries were performed under the senior author’s direct supervision. This study was approved by the ethics board of the Friedrich Alexander University of Erlangen Nuremberg (registration number: 21-433-Br) and the local ethic committee of Rhineland-Palatinate (registration number: 2021-16096). Patients who either received single or combined PPF reconstruction with a minimum rotation arc of 45 degrees were included in the study. Combined PPFs were defined as “double PPF” if two perforator propeller flaps were used whereas those combined with any kind of regional flap were assigned to the group of “PPF plus regional flap”.

We utilized patients’ digital charts to collect data on individual characteristics, flap surgery, risk factors as well as postoperative complications. Risk factors considered were diabetes, arterial hypertension, peripheral artery disease, coronary heart disease, coagulation disorders, prior thrombotic events, obesity (BMI > 30 kg/m^2^), radiation therapy, chemotherapy, and smoking. All risk factors as well as postoperative complications were assessed separately before creating a dichotomous variable. Furthermore, postoperative complications requiring surgical treatment were considered as a “major complication” whereas those manageable by conservative therapy were considered as a “minor complication”. Partial flap loss was defined as a flap necrosis of at least five percent, which did not result in a total flap removal, while “total flap loss” was defined as a flap loss of more than 50 percent leading to reconstructive failure and, thus, total removal of the flap. Additionally, we recorded the total time of hospitalization as well as the total amount of surgeries related to the specific type of PPF reconstruction surgery (single or combined PPF). We chose “major complication” as the primary outcome variable since, by definition, all kinds of major complications resulted in additional surgeries and, hence, a more complex course of disease.

### 2.2. Methods

#### Flap Harvesting

**Figure 1:**
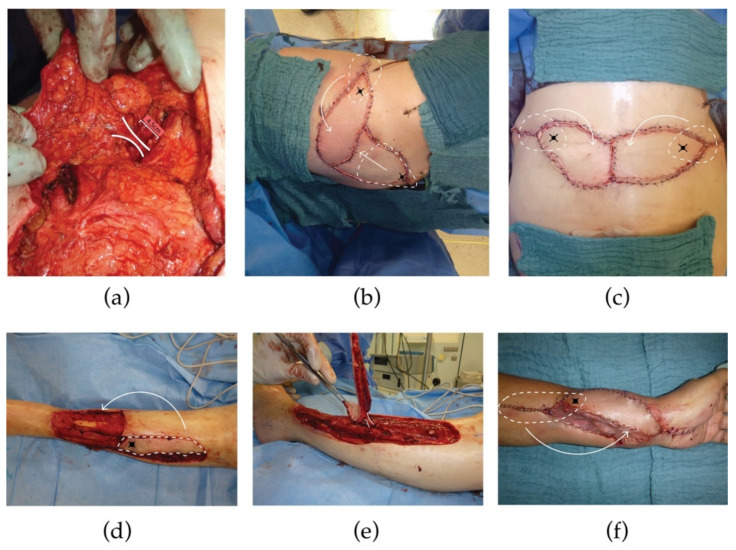
Principle of flap harvesting. (**a**–**c**) demonstrates PPF reconstruction on the trunk. (**a**) Meticulous dissection of the perforator. We aimed for a minimum length of 3 to 5 centimeters to avoid vascular complications; (**b**) PPF plus perforator-based VY advancement flap. Black crosses indicate perforators of the flaps. After skin incision and dissection of surrounding tissue, the PPF was rotated (curved arrow) into the defect, while the VY advancement flap was transposed (straight arrow) into the defect; (**c**) Double PPF: Black crosses indicate pivot points given by the perforators. Those were marked together with the regions of flap harvesting (white dashed lines) prior to surgery. After skin incision and dissection of surrounding tissue, flaps were rotated into the defect (white dashed lines); (**d**,**e**) Demonstrates PPF reconstruction of the lower extremity; (**f**) Demonstrates PPF reconstruction of the upper extremity (Abbreviations: PPF, perforator propeller flap).

Prior to surgery, relevant perforating vessels were identified by either using a handheld Doppler device (2008−2014) or duplex ultrasound (2015−2021) [18,19]. Preoperative planning of flap design, flap dimensions as well as flap orientation were adapted to the size, location, and vascular territory of the defect. If appropriate, combined PPF designs were considered to ensure tension-free donor-site closure. Flap dissection was performed with subsequent localization and preservation of significant perforators. It was conducted in a manner for which the chance of converting to a conventional random pattern flap was preserved. This procedure was intended as a rescue strategy if suitable perforators were absent or turned out to be insufficient. However, conversion to random pattern skin flaps was not necessary. Since the vascular pedicle represents the central axis of rotation and its length is inversely proportional to the critical angle of twisting, we aimed, whenever possible, for a minimum pedicle length of 3 to 5 centimeters to ensure adequate flap perfusion (Figure 1a) [20,21,22]. To verify sufficient blood flow, we intraoperatively clamped all preserved perforators except for the dominant one prior to complete flap harvest. Evaluation of the dominant perforator followed in terms of caliber size, pulsatility, and morphology. The flap was then rotated into the defect either in a clockwise or counterclockwise direction, depending on which method led to lower twisting of the pedicle. Flap perfusion was assessed clinically. In addition to clinical flap assessment, indocyanine green fluorescence angiography was performed for objective flap perfusion assessment in selected cases [23]. Figure 1 visualizes the principle of flap harvesting in further detail.

### 2.3. Statistical Analysis

Separately for single PPF and combined PPF reconstruction surgery, we estimated the prevalence of major complications. To identify whether characteristics among the approaches differ, we conducted Wilcoxon–Mann–Whitney tests on continuous variables of defect size, PPF size, total days of hospitalization, age, total number of surgeries, and operation time against a patients’ type of perforator propeller reconstruction surgery as well as Fisher’s exact tests on categorical variables of sex, major complications, and existing risk factors. Furthermore, separately for the groups of (I) all PPFs, (II) single PPFs, and (III) combined PPFs, we regressed major complications onto individuals’ defect size, propeller flap size, and operation time. Those analyses were applied to assess the effect an independent variable had on the outcome depending on the type of surgical intervention. Finally, we regressed the type of reconstructive flap surgery on major complications. All regression models were performed by employing univariable binary logistic regression analyses. We considered an error probability of *p* ≤ 0.05 as statistically significant. Statistical analyses were conducted using R (version 3.6.1, open source). Visualization was performed wherever necessary using RStudio (version 1.1.456, RStudio PBC, Boston, MA, USA) and Adobe Illustrator CS6 (Adobe Systems Incorporated, Mountain View, San Jose, CA, USA).

## 3. Results

### 3.1. Overall Sample Characteristics

Of 86 patients (38 female, 48 male) that underwent PPF reconstruction surgery between 2008 and 2021, 69 patients received single PPFs (69 flaps) whereas 17 patients received combined PPF reconstruction (28 PPFs). Mean age of patients was 56.7 years ranging from 4 years to 88 years. Twenty-nine patients (33.7%) presented with risk factors for postoperative complications prior to surgery (Table 2). Tumor and pressure ulcer were the most common indications for PPF reconstruction and accounted for more than 66.3% of all indications. A total of 62 (72.1%) reconstructions were performed on the trunk, 18 (20.9%) on the lower extremity, and 6 (7.0%) on the upper extremity. Of all single PPF reconstructions performed, 47 (68.1%) were performed on the trunk, 16 (23.2%) on the lower extremity and 6 (8.7%) on the upper extremity. Among combined PPF reconstructions 15 (88.2%) were performed on the trunk and 2 (11.8%) on the lower extremity. Mean operation time was 178 minutes, ranging from 80 to 480 minutes. Mean defect size was 117.8 ± 88.6 cm^2^ ranging from 12 to 504 cm^2^, while mean PPF size was 137.3 ± 85.1 cm^2^ ranging from 24 to 532 cm^2^. Overall, major complications occurred in 27 patients (31.4%). Partial flap loss occurred in 5 patients (7.2%) and total flap loss in 5 patients (5.8%). In summary, primary reconstruction was successful in 60 out of 69 patients (87.0%) with single PPFs and in 16 out of 17 patients (94.1%) with combined PPFs by the time of discharge from our department. Patient and flap characteristics are shown in detail in Table 2.

### 3.2. Comparison of Single PPFs and Combined PPFs

**Figure 2:**
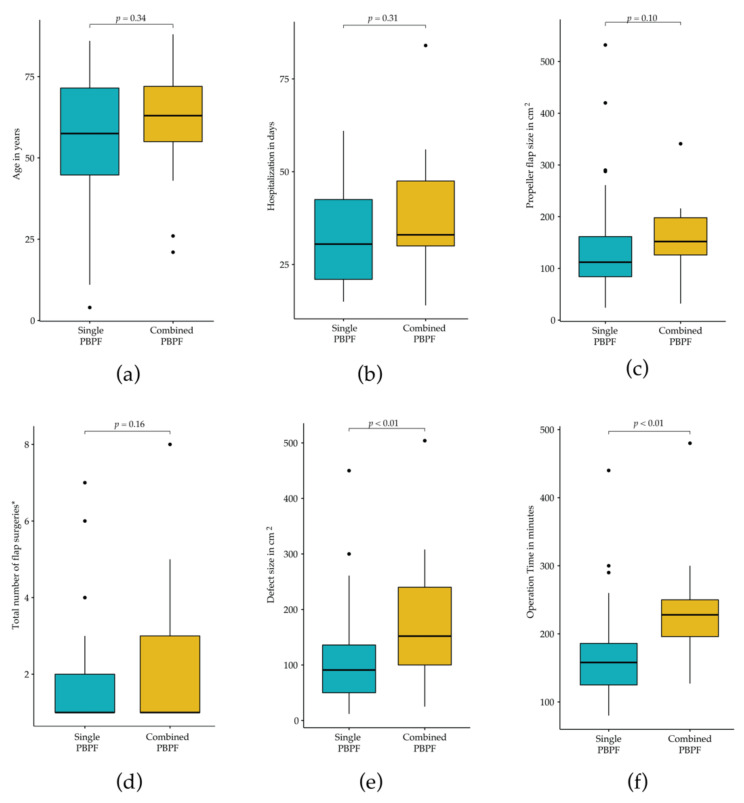
Wilcoxon–Mann–Whitney test on continuous variables. (**a**) Age in years; (**b**) Total hospitalization in days; (**c**) Perforator propeller flap size in cm^2^; (**d**) Total number of surgeries starting with PPF surgery; (**e**) Defect size in cm^2^; (**f**) Operation time in minutes (Abbreviations: PPF, perforator propeller flap).

Both groups had similar characteristics in terms of age (*p* = 0.34), sex (*p* = 0.79), PPF size (*p* = 0.10), flap rotation (*p* = 0.53), total number of surgeries associated with flap surgery (*p* = 0.16), and days of hospitalization (*p* = 0.31) (Table 2, Figure 2). Risk factors were more frequent among patients undergoing combined PPF reconstruction (58.8% vs. 27.5%; *p* = 0.02). Furthermore, tumors and pressure ulcers were the main causes for PPFs in both groups with a shared proportion of 63.8% among single PPFs and 76.4% among combined PPFs, respectively (Table 2). While most reconstructions were performed on the trunk, this proportion was 88.2% among combined PPFs and 68.1% among single PPFs. Lumbar artery perforator flaps (14.5% of single PPFs vs. 28.6% of combined PPFs), inferior gluteal artery perforator flaps (13.0% of single PPFs vs. 21.4% of combined PPFs), and superior gluteal artery perforator flaps (11.6% of single PPFs vs. 21.4% of combined PPFs) were the most common flaps used among both groups (Table 3). Defect sizes of combined PPFs were significantly larger than they were among single PPFs (178.2 ± 73.8 cm^2^ vs. 103.0 ± 73.5 cm^2^, *p* < 0.01) and mean operation time was significantly longer among combined PPFs than those among single PPFs (164 ± 59 vs. 233 ± 76 minutes, *p* < 0.01) (Figure 2). However, there were no significant differences in the occurrence of major complications (31.9% (single PPFs) vs. 29.4% (combined PPFs), *p* = 0.32) as well as complete flap losses (5.8% (single PPFs) vs. 5.9% (combined PPFs), *p* = 0.99). Table 2 provides a detailed summary of the results.

### 3.3. Univariable Binary Logistic Regression

We included independent variables we thought could be used for clinical decision-making due to the prediction of major complications and that could be relatively easy to be ascertained by clinicians without needing to undertake complex imaging or laboratory measurements. We included independent variables of operation time, type of intervention, PPF size, and defect size. Defect size was included in our analysis as broad categories (<50 cm^2^ [reference], 50−99 cm^2^, 100−149 cm^2^, 150−199 cm^2^, and >200 cm^2^) under the rationale that defect sizes are rather approximations than precise measurements due to complex geometry and the unclear extent of many defects. Regression analyses revealed that the type of intervention (OR: 0.95, 95% CI: 0.27−2.93; *p* = 0.29), the operation time (OR: 1.00, 95% CI: 1.00−1.01; *p* = 0.25) and the PPF size (OR: 1.00, 95% CI: 0.99−1.01; *p* = 0.15) had no significant impact on the occurrence of major complications (Table 4). However, we found a substantial higher probability of having major complications among defects larger than 200 cm^2^ (OR: 8.50, 95% CI: 1.51−70.24; *p* < 0.01). To find out whether this was true for both groups, we conducted subgroup analyses separately for single PPFs and combined PPFs. Subgroup analyses revealed that there was a significant increase of major complications in defects larger than 100 cm^2^ only in the group of single PPFs. However due to little data, confidence intervals of the subgroup analyses were disproportionately large. Consequently, we conducted an additional univariable binary logistic regression analysis, which only considered defect sizes larger than 100 cm^2^. This analysis verified prior results. Defects larger than 100 cm^2^ were significantly associated with an increased risk of having major complications among single PPFs (OR: 2.82, 95% CI: 1.01−8.36; *p* = 0.05). In contrast, there was no increased risk among combined PPFs (OR: 0.30, 95% CI: 0.02−3.37; *p* = 0.32). In fact, defects larger than 100 cm^2^ only proved to be a significant predictor of major complications among single PPFs. Results of the regression analyses are shown in detail in Table 4.

## 4. Discussion

While complete flap losses were rare events, we found a considerable prevalence of major complications of 31.9% among patients that received single PPFs and 29.4% among patients that received combined PPFs. Individual characteristics of sex, age, flap location, flap size, arc of flap rotation, total number of surgeries, and total flap loss were similar among the groups. Notably, defect sizes of combined PPF reconstructions were substantially larger than they were among single PPF reconstructions. While the operation time, the type of flap surgery, and the PPF size were not associated with the probability of having an increase in major complications, the defect size was. Interestingly, this was true for defects larger than 100 cm^2^ among single PPFs but not among combined PPFs. Our findings have several important implications.

First, with a total flap loss of less than six percent, we have shown that combined PPFs were a reliable and promising reconstructive approach in the reconstruction of large soft tissue defects, which has thus far received little attention from a research perspective.

Second, the propeller flap size, the operation time as well as the type of flap surgery were not associated with a higher risk of having major complications among perforator propeller flaps. This, however, is interesting since flap size and operation time are known predictors for postoperative complications in free and regional flap surgery [24,25]. One possible explanation why we did not observe more major complications among larger flaps might be because most PPFs were performed on the trunk where perforasomes are usually quite large and, thus, may guarantee adequate flap perfusion. Another explanation could be the use of indocyanine green fluorescence angiography additional to clinical flap perfusion assessment in critical cases. Prior research implies that this method can reduce the rate of partial flap necrosis and improves flap survival through detection of insufficiently perfused flap areas [23,26,27]. However, further studies are needed to understand the underlaying factors of our findings. Additionally, our results showed that combined PPFs were as safe as single PPFs regardless of the significantly larger defect size among combined PPFs.

Third, a defect size of more than 100 cm^2^ was a significant predictor of major complications only among single PPFs, suggesting that surgeons should prefer combined PPFs for the reconstruction of large soft tissue defects. This is an important finding since free flap surgery constitutes the alternative approach that might require complex surgical procedures, such as arteriovenous loops in order to provide adequate recipient vessels [28].

Fourth, the prevalence of major complications among combined PPF surgery is comparable to that reported among free flap surgery indicating that both options should be weighed carefully against each other when it comes to the reconstruction of large soft tissue defects [29,30,31]. For instance, due to microvascular anastomosis, free flaps require a longer operation time as well as longer postoperative immobilization than PPFs. Thus, the use of PPFs might decrease morbidity and mortality in selected patients [32,33,34].

Despite PPFs gaining popularity in recent years, research has mainly focused on single PPFs which, however, have proven to be a reliable method with a low complication rate [35,36]. Combined PPFs may allow reconstruction of extended defects and therefore, extent reconstructive possibilities [37]. For instance, Scaglioni and colleagues reported successful reconstruction of a large gluteal defect by a combination of two PPFs and a VY advancement flap [38]. This supports our experience as we could demonstrate sufficient reconstruction of the posterior trunk by a combination of two PPFs. Due to scarcity of recipient vessels, this body region is often unsuited for free flap reconstruction [8]. However, the combined PPF technique still is considered as a rescue strategy whenever established reconstructive approaches were not suitable. Given that, we suggest further prospective studies to specifically investigate the safety of combined PPFs in the reconstruction of large soft tissue defects and to assess the implementation of combined PPFs in the daily routine of reconstructive surgeons.

Although our results are promising, this study has several limitations. First, due to the retrospective design and small number of cases, this study might be underpowered. Second, we could only collect data documented in a patient’s digital chart. Third, we used major complication rates of free flaps that were reported in the literature for comparison. Patients included in those studies might not reflect our patients, which is why further studies should aim for a prospective approach that include both, PPF and free flap surgery, to avoid possible bias. Fourth, most of the patients included in our study received PPF reconstructions on the trunk, which is why our results mainly account for this region of the body. Even though it is likely that our results can be applied to any region of the body, we strongly recommend further studies that specifically focus on the comparison of single and combined PPFs on the upper and lower extremity.

## 5. Conclusions

Our study is the first to compare single and combined perforator propeller flaps in terms of major postoperative complications. We could show that combined PPFs are not just a reliable reconstructive option, but eventually presented fewer major complications than single PPFs in the reconstruction of large soft tissue defects. Furthermore, major complication rates of combined PPFs were comparable to those among free flap surgery. Consequently, combined PPFs should be considered as a first line technique in the reconstruction of large soft tissue defects of the trunk. However, since combined PPFs require a flexible surgical strategy and intraoperative decision-making is sometimes challenging, we recommend proper training before implementing combined PPFs in a daily routine to achieve reliable results.

## Figures and Tables

**Table 1:** Articles ^1^ reporting the use of combined perforator propeller flaps.

Title	Author	Year	No. of Patients	Body Region
Dual Reconstruction of Lumbar and Gluteal Defects with Freestyle Propeller Flap and Muscle Flap	Ellabban et al. [13]	2021	18	Trunk/Gluteal
Lumbar Perforator Flaps for Coverage of Extensive Defects With Osteomyelitis	Schaffer et al. [14]	2021	7	Trunk
Perforator-Based Flaps for Defect Reconstruction of the Posterior Trunk	Hernekamp et al. [9]	2021	36	Trunk
Use of the Propeller Lumbar Perforator Flap: A Series of 32 Cases	Falinower et al. [15]	2020	31	Trunk
The SCIP propeller flap: Versatility for reconstruction of locoregional defect	Boissière et al. [16]	2019	56	Trunk
Freestyle multiple propeller flap reconstruction (jigsaw puzzle approach) for complicated back defects	Park et al. [17]	2015	18	Trunk

^1^ Case reports as well as articles that did not include combined perforator propeller flaps were excluded.

**Table 2:** Patient characteristics.

Characteristic	Total	Single PPF	Combined PPF	*p*-Value
No. of patients (%)	86	69 (80.2)	17 (19.8)	
Combined PPF, No. (%)	17 (19.8)			
Double PPF			11 (64.7)	
PPF plus regional flap			6 (35.3)	
Sex, No. (%)				0.79
Female	38 (44.2)	30 (43.5)	8 (47.1)	
Male	48 (55.8)	39 (56.5)	9 (52.9)	
Mean age [years] (SD, range)	56.7 (19.7, 4−88)	55.7 (20.0, 4−86)	60.8 (18.6, 21−88)	0.34
Risk factors ^1^ present, No. (%)	29 (33.7)	19 (27.5)	10 (58.8)	0.02
Defect etiology (%)				
Burn injury	1 (1.2)	1 (1.4)	0 (0.0)	0.36
Pressure ulcer	13 (15.1)	10 (14.5)	3 (17.6)	0.72
Infection	8 (9.3)	6 (8.7)	2 (11.8)	0.65
Trauma	6 (7.0)	4 (5.8)	2 (11.8)	0.39
Tumor	44 (51.2)	34 (49.3)	10 (58.8)	0.59
Other	14 (16.3)	14 (20.3)	0 (0.0)	
Defect size in [cm^2^] (SD, range)	117.8 (88.6, 12−504)	103.0 (73.5, 12−450)	178.2 (73.8, 25−504)	<0.01
PPF size [cm^2^] (SD, range)	137.3 (85.1, 24−532)	132.8 (88.0, 24−532)	155.2 (71.6, 32−341)	0.10
Flap location (%)				
Trunk	62 (72.1)	47 (68.1)	15 (88.2)	0.13
Lower limb	18 (20.9)	16 (23.2)	2 (11.8)	0.50
Upper limb	6 (7.0)	6 (8.7)	0 (0.0)	0.34
Operation time [min] (SD, range)	177.6 (68.0, 80−480)	164.0 (59.0, 80−440)	232.9 (75.6, 127−480)	<0.01
Flap rotation [degree] (SD, range)	149.9 (35.0, 50−180)	147.1 (37.1, 50−180)	156.4 (29.8, 90−180)	0.53
Number of surgeries ^2^ (SD, range)	1.7 (1.4, 1−8)	1.5 (1.1, 1−7)	2.3 (2.0, 1−8)	0.16
Major complications ^3^ (%)	27 (31.4)	22 (31.9)	5 (29.4)	0.32
Flap loss (%)				
Partial	5 (7.2)	5 (7.2)	0 (0.0)	0.56
Complete	5 (5.8)	4 (5.8)	1 (5.9)	0.99
Total hospitalization [days] (SD, range)	34.7 (15.7, 14−84)	32.5 (13.6, 15−61)	39.7 (19.7, 14−84)	0.31

Abbreviations: PPF, perforator propeller flap; ^1^ includes risk factors of diabetes, arterial hypertension, peripheral artery disease, coronary heart disease, coagulation disorders, prior thrombotic events, obesity (BMI > 30 kg/m^2^), radiation therapy, chemotherapy, and smoking; ^2^ with PPF surgery being the first surgery counted; ^3^ includes postoperative complications that required surgical revision during the time of hospitalization.

**Table 3:** Flap distribution.

Characteristics	Total	Single PPF	Combined PPF
No. of PPFs (%)	97	69 (71.1)	28 (28.9)
PPF type, No. (%)			
Adductor perforator	5 (5.2)	3 (4.3)	2 (7.1)
ALT	5 (5.2)	4 (5.8)	1 (3.6)
ATA	3 (3.0)	3 (4.3)	0 (0.0)
AIA	3 (3.0)	3 (4.3)	0 (0.0)
PTA	5 (5.2)	5 (7.2)	0 (0.0)
Brachial artery	4 (4.1)	4 (5.8)	0 (0.0)
DICAP	7 (7.3)	4 (5.8)	3 (10.7)
IGAP	15 (15.5)	9 (13.0)	6 (21.4)
LICAP	3 (3.0)	2 (2.9)	1 (3.6)
Radial artery	3 (3.0)	3 (4.3)	0 (0.0)
SGAP	14 (14.4)	8 (11.6)	6 (21.4)
Lateral genicular artery	1 (1.0)	1 (1.4)	0 (0.0)
LAP	18 (18.6)	10 (14.5)	8 (28.6)
Posterior thigh perforator	2 (2.0)	2 (2.9)	0 (0.0)
Profound femoral artery	3 (3.0)	3 (4.3)	0 (0.0)
Pudendal artery	2 (2.0)	1 (1.4)	1 (3.6)
Thoracoacromial artery	2 (2.0)	2 (2.9)	0 (0.0)
Trapezius perforator	2 (2.0)	2 (2.9)	0 (0.0)

Abbreviations: PPF, perforator propeller flap; ALT, anterior lateral thigh; ATA, anterior tibial artery; AIA, anterior intercostal artery; PTA, posterior tibial artery; DICAP, dorsal intercostal artery perforator; IGAP, inferior gluteal artery perforator; LICAP, lateral intercostal artery perforator; SGAP, superior gluteal artery perforator; LAP, lumbar artery perforator.

**Table 4:** Univariable binary logistic regressions of major complications among 86 patients.

	Total PPF (*n* = 86)		Single PPF (*n* = 69)		Combined PPF (*n* = 17)	
	Odds Ratio		Odds Ratio		Odds Ratio	
Characteristics	(95% CI)	*p*-Value	(95% CI)	*p*-Value	(95% CI)	*p*-Value
Intervention						
Single PPF	1 [Reference]					
Combined PPF	0.95 (0.27−2.93)	0.29				
Operation time (min)	1.00 (1.00−1.01)	0.25	1.00 (0.99−1.01)	0.80	1.01 (1.00−1.04)	0.33
PPF size (cm^2^)	1.00 (0.99−1.01)	0.15	1.00 (1.00−1.01)	0.50	1.01 (1.00−1.03)	0.11
Defect size (cm^2^)						
<50	1 [Reference]		1 [Reference]		1 [Reference]	
50−99	3.97 (0.80−29.48)	0.17	6.85 (1.00−137.99)	0.13	1.00 (0.01−69.47)	0.99
100−199	4.25 (0.97−29.89)	0.08	10.00 (1.63−194.65)	0.05	0.17 (0.01−6.53)	0.58
>200	8.50 (1.51−70.24)	<0.01	32.00 (3.05−850.50)	0.01	0.50 (0.01−17.47)	0.99
Defect size (cm^2^)						
<100	1 [Reference]		1 [Reference]		1 [Reference]	
>100	1.88 (0.74−4.91)	0.18	2.82 (1.01−8.36)	0.05	0.30 (0.02−3.37)	0.32

Abbreviations: PPF = perforator propeller flap.

## Data Availability

Data sharing is not applicable to this article.

## References

[1] Baumann D.P., Butler C.E. (2013). Soft tissue coverage in abdominal wall reconstruction. Surg. Clin. N. Am..

[2] Falkner F., Thomas B., Haug V., Nagel S.S., Vollbach F.H., Kneser U., Bigdeli A.K. (2021). Comparison of pedicled versus free flaps for reconstruction of extensive deep sternal wound defects following cardiac surgery: A retrospective study. Microsurgery.

[3] Xiong L., Gazyakan E., Kremer T., Hernekamp F.J., Harhaus L., Saint-Cyr M., Kneser U., Hirche C. (2016). Free flaps for reconstruction of soft tissue defects in lower extremity: A meta-analysis on microsurgical outcome and safety. Microsurgery.

[4] Bigdeli A.K., Thomas B., Falkner F., Radu C.A., Gazyakan E., Kneser U. (2020). Microsurgical reconstruction of extensive lower extremity defects with the conjoined parascapular and latissimus dorsi free flap. Microsurgery.

[5] Beier J.P., Horch R.E., Kneser U. (2013). Bilateral pre-expanded free TFL flaps for reconstruction of severe thoracic scar contractures in an 8-year-old girl. J. Plast. Reconstr. Aesthetic Surg..

[6] Wanjala N.F., Dan K. (2020). Local/regional flaps for extensive abdominal wall defects: Case series. Int. J. Surg. Case Rep..

[7] Ebehr B., Wagner J.M., Ewallner C., Eharati K., Elehnhardt M., Daigeler A. (2016). Reconstructive Options for Oncologic Posterior Trunk Defects: A Review. Front. Oncol..

[8] Hernekamp J.-F., Cordts T., Kremer T., Kneser U. (2020). Perforator-Based Flaps for Defect Reconstruction of the Posterior Trunk. Ann. Plast. Surg..

[9] Hallock G.G. (2006). The Propeller Flap Version of the Adductor Muscle Perforator Flap for Coverage of Ischial or Trochanteric Pressure Sores. Ann. Plast. Surg..

[10] Yang C.-H., Kuo Y.-R., Jeng S.-F., Lin P.-Y. (2011). An Ideal Method for Pressure Sore Reconstruction: A freestyle perforator-based flap. Ann. Plast. Surg..

[11] Pignatti M., Ogawa R., Hallock G.G., Mateev M., Georgescu A.V., Balakrishnan G., Ono S., Cubison T.C.S., D’arpa S., Koshima I. (2011). The “Tokyo” Consensus on Propeller Flaps. Plast. Reconstr. Surg..

[12] Yu S., Zang M., Xu L., Zhao Z., Zhang X., Zhu S., Chen B., Ding Q., Liu Y. (2016). Perforator Propeller Flap for Oncologic Reconstruction of Soft Tissue Defects in Trunk and Extremities. Ann. Plast. Surg..

[13] Ellabban M.A., Wyckman A., Abdelrahman I., Steinvall I., Elmasry M. (2021). Dual Reconstruction of Lumbar and Gluteal Defects with Freestyle Propeller Flap and Muscle Flap. Plast. Reconstr. Surg. Glob. Open.

[14] Schaffer C., Guillier D., Raffoul W., di Summa P.G. (2021). Lumbar Perforator Flaps for Coverage of Extensive Defects With Osteomyelitis. Ann. Plast. Surg..

[15] Falinower H., Herlin C., Laloze J., Bodin F., Kerfant N., Chaput B. (2020). Use of the Propeller Lumbar Perforator Flap: A Series of 32 Cases. Plast. Reconstr. Surg. Glob. Open.

[16] Boissière F., Luca-Pozner V., Vaysse C., Kerfant N., Herlin P.C., Chaput P.B. (2019). The SCIP propeller flap: Versatility for reconstruction of locoregional defect. J. Plast. Reconstr. Aesthetic Surg..

[17] Park S.W., Oh T.S., Eom J.S., Sun Y.C., Suh H.S., Hong J.P. (2015). Freestyle Multiple Propeller Flap Reconstruction (Jigsaw Puzzle Approach) for Complicated Back Defects. J. Reconstr. Microsurg..

[18] Thomas B., Haug V., Falkner F., Arras C., Nagel S.S., Boecker A., Schmidt V.J., Kneser U., Bigdeli A.K. (2021). A single-center retrospective comparison of Duplex ultrasonography versus audible Doppler regarding anterolateral thigh perforator flap harvest and operative times. Microsurgery.

[19] Daigeler A., Schubert C., Hirsch T., Behr B., Lehnhardt M. (2018). Colour duplex sonography and “Power-Duplex” in Perforator Surgery—Improvement of patients safety by efficient planning. Handchir. Mikrochir. Plast. Chir..

[20] Wong C.-H., Cui F., Tan B.-K., Liu Z., Lee H.-P., Lu C., Foo C.-L., Song C. (2007). Nonlinear Finite Element Simulations to Elucidate the Determinants of Perforator Patency in Propeller Flaps. Ann. Plast. Surg..

[21] Selvaggi G., Anicic S., Formaggia L. (2006). Mathematical explanation of the buckling of the vessels after twisting of the microanastomosis. Microsurgery.

[22] Topalan M., Bilgin S.S., Ip W., Chow S. (2003). Effect of torsion on microarterial anastomosis patency. Microsurgery.

[23] Bigdeli A.K., Thomas B., Falkner F., Gazyakan E., Hirche C., Kneser U. (2020). The Impact of Indocyanine-Green Fluorescence Angiography on Intraoperative Decision-Making and Postoperative Outcome in Free Flap Surgery. J. Reconstr. Microsurg..

[24] Lin P., Kuo P., Kuo S.C.H., Chien P., Hsieh C. (2020). Risk factors associated with postoperative complications of free anterolateral thigh flap placement in patients with head and neck cancer: Analysis of propensity score-matched cohorts. Microsurgery.

[25] Peng P., Dong Z., Wei J., Liu L., Luo Z., Zheng L. (2021). Risk factors related to the partial necrosis of the posterior tibial artery perforator-plus fasciocutaneous flap. Eur. J. Trauma Emerg. Surg..

[26] Jakubietz R.G., Schmidt K., Bernuth S., Meffert R.H., Jakubietz M.G. (2019). Evaluation of the Intraoperative Blood Flow of Pedicled Perforator Flaps Using Indocyanine Green-fluorescence Angiography. Plast. Reconstr. Surg. Glob. Open.

[27] Kneser U., Beier J.P., Schmitz M., Arkudas A., Dragu A., Schmidt V.J., Kremer T., Horch R.E. (2013). Zonal perfusion patterns in pedicled free-style perforator flaps. J. Plast. Reconstr. Aesthetic Surg..

[28] Henn D., Wähmann M.S.T., Horsch M., Hetjens S., Kremer T., Gazyakan E., Hirche C., Schmidt V.J., Germann G., Kneser U. (2019). One-Stage versus Two-Stage Arteriovenous Loop Reconstructions: An Experience on 103 Cases from a Single Center. Plast. Reconstr. Surg..

[29] Farquhar D.R., Masood M.M., Pappa A.K., Patel S.N., Hackman A.T.G. (2018). Predictors of Adverse Outcomes in Free Flap Reconstruction: A Single-Institution Experience. Otolaryngol. Neck Surg..

[30] Le Nobel G.J., Higgins K.M., Enepekides D.J. (2012). Predictors of complications of free flap reconstruction in head and neck surgery: Analysis of 304 free flap reconstruction procedures. Laryngoscope.

[31] Classen D.A., Ward H. (2006). Complications in a Consecutive Series of 250 Free Flap Operations. Ann. Plast. Surg..

[32] Guo F., Shashikiran T., Chen X., Yang L., Liu X., Song L. (2015). Clinical features and risk factor analysis for lower extremity deep venous thrombosis in Chinese neurosurgical patients. J. Neurosci. Rural. Pract..

[33] Brogan E., Langdon C., Brookes K., Budgeon C., Blacker D. (2014). Respiratory Infections in Acute Stroke: Nasogastric Tubes and Immobility are Stronger Predictors than Dysphagia. Dysphagia.

[34] Chou C.-L., Lee W.-R., Yeh C.-C., Shih C.-C., Chen T.-L., Liao C.-C. (2015). Adverse Outcomes after Major Surgery in Patients with Pressure Ulcer: A Nationwide Population-Based Retrospective Cohort Study. PLoS ONE.

[35] Vitse J., Bekara F., Bertheuil N., Sinna R., Chaput B., Herlin C. (2016). Perforator-based propeller flaps reliability in upper extremity soft tissue reconstruction: A systematic review. J. Hand Surg. Eur. Vol..

[36] Iida T., Yoshimatsu H., Koshima I. (2017). Reconstruction of Anterolateral Thigh Defects Using Perforator-Based Propeller Flaps. Ann. Plast. Surg..

[37] Arco G., Horch R.E., Arkudas A., Dragu A., Bach A.D., Kneser U. (2009). Double pedicled perforator flap to close flank defects: An alternative for closure of a large lumbar defect after basalioma excision--A case report and review of the literature. Ann. Plast. Surg..

[38] Scaglioni M.F., Grufman V., Meroni M., Fritsche E. (2020). Soft tissue coverage of a total gluteal defect with a combination of perforator-based flaps: A case report. Microsurgery.

